# Tangnaikang Alleviates Hyperglycemia and Improves Gut Microbiota in Diabetic Mice

**DOI:** 10.1155/2021/1089176

**Published:** 2021-11-25

**Authors:** Liping Zhang, Fen Wang, Hualiang He, Tingting Jiao, Lili Wu

**Affiliations:** ^1^Endocrinology Department, Beijing University of Chinese Medicine Third Affiliated Hospital, Beijing, China; ^2^Emergency Department, Armed Police Force Beijing General Hospital, Beijing, China; ^3^Beijing University of Chinese Medicine, Beijing, China; ^4^Institute of Health Science, Beijing University of Chinese Medicine, Beijing, China

## Abstract

Dysregulation of gut microbiota contributes to the development of type 2 diabetes. To investigate the antidiabetic effect of Tangnaikang and its regulation of gut microbiota in diabetic KKAy mice, a type 2 diabetes mouse model was established by feeding KKAy mice with a high-fat diet (HFD) for 2 weeks. The diabetic KKAy mice were treated with vehicle, Acarbose, or different doses of Tangnaikang once a day for 8 weeks. The fasting plasma glucose (FPG) levels and bodyweights were measured weekly. The fecal and blood samples were collected 8 weeks after treatment. The 16s rRNA sequencing and bioinformatics analysis were conducted to explore the effects of Tangnaikang treatment on the richness, diversity, and relative abundance of gut microbiota. Compared with other treatments, high-dose Tangnaikang (4.68 g/kg) significantly reduced FPG levels while elevating bodyweights in model mice. Compared with saline treatment, different doses of Tangnaikang significantly increased gut microbial species richness and diversity. Linear discriminant analysis effect size identified potential bacterial biomarkers associated with Tangnaikang treatment. Relative abundance analysis revealed that Tangnaikang treatment modulated the abundance of gut bacteria at the class and genus levels, such as *Bacilli*, *Lactobacillus,* and *Alistipes*. The principal component analysis demonstrated that, compared with the samples of the high-dose group, the samples of medium-dose and low-dose groups were closer to those of the model group. Tangnaikang alleviated hyperglycemia and improved the composition and abundance of gut microbiota in diabetic KKAy mice.

## 1. Introduction

Diabetes mellitus (DM) is a group of metabolic disorders characterized by high blood glucose levels, and type 2 diabetes (T2D) accounts for about 90% of all diabetes cases worldwide [1]. International Diabetes Federation (IDF) has estimated that there were 451 million people with diabetes worldwide in 2017 and predicted that this number will increase to 693 million by 2045 [2]. There is an urgent need to develop effective therapeutic strategies to counteract these rising trends.

Gut microbiota refers to the community of microorganisms residing in the gut. Recent studies have identified that changes in the quantity and diversity of gut microbiota play an important role in the development of T2D [3, 4]. Accumulating data have suggested that compositional and functional changes in gut microbiota affect host glucose homeostasis. For example, fecal transplants from mice with glucose intolerance induce glucose intolerance in healthy germ-free mice [5]. On the other hand, fecal transplants from lean donors improve gut microbial diversity and insulin sensitivity in patients with metabolic syndrome [6]. Thus, modulation of gut microbiota is a promising strategy to prevent or reverse the development of T2D [7].

Traditional Chinese medicine has been shown to ameliorate T2D by regulating gut microbiota in animal studies and clinical trials [8, 9]. Tangnaikang is a traditional Chinese medicine formula composed of herbal medicines *Panax ginseng* C.A. Meyer, *Ligustrum lucidum* Ait., *Prunella vulgaris* L., *Saururus chinensis* (Lour.) Baill, and *Psidium guajava* Linn. Studies have shown that Tangnaikang ameliorates glucose intolerance and insulin resistance in prediabetic SHR/cp rats and obese Zucker rats [10, 11]. The main Chinese herbs in Tangnaikang, such as *Fructus Ligustri Lucidi* and *Radix Ginseng*, can regulate gut microbiota structure and diversity in mouse models [12, 13]. However, the involvement of gut microbiota in the antidiabetic effect of Tangnaikang remains unknown.

In this study, we evaluated the antidiabetic role of Tangnaikang in high-fat diet- (HFD-) induced diabetic KKAy mice. Through 16S rRNA sequencing and bioinformatics analysis, we investigated the alterations in gut microbiota in response to Tangnaikang treatment. Our results suggest that improving gut microbial balance is involved in the antidiabetic role of Tangnaikang.

## 2. Materials and Methods

### 2.1. Animals

KKAy mice are an animal model of spontaneous diabetes mellitus. The animal study was approved by the Ethics Committee of the Institute of Basic Theory of Chinese Medicine, China Academy of Chinese Medical Sciences (approval #SYXK (Jing) 2016-0021, 2019-067; Beijing, China). All procedures were conducted following the guidelines for the Care and Use of Laboratory Animals of China Academy of Chinese Medical Sciences. Specific-pathogen-free-grade male KKAy mice and C57BL mice (10-week-old, 35.5 ± 3.0 g) were purchased from HFK Bio-Technology (Beijing, China). The animals were maintained in a 12 h light/dark cycle at 21 ± 2°C and 60 ± 10% humidity with free access to water and food. After a week of acclimation, the C57BL mice (*n* = 13) were fed a chow diet; the KKAy mice were fed HFD (HFK Bio-Technology) for two weeks. KKAy mice with random blood glucose levels ≥13.9 mmol/L [14] were considered T2D model mice (*n* = 65).

### 2.2. Tangnaikang Treatment

Tangnaikang granules composed of *Panax ginseng* C.A. Meyer, *Ligustrum lucidum* Ait.*, Prunella vulgaris* L.*, Saururus chinensis* (Lour.) Baill., and *Psidium guajava* Linn. were obtained from the Pharmaceutical Factory of Beijing University of Chinese Medicine (Beijing, China). 1 g granule is equivalent to 3.61 g crude drugs. For animal treatment, Tangnaikang granules were dissolved in distilled water, sonicated for 30 min, and stored at 4°C until use.

C57BL mice (*n* = 13) were assigned to the control group. Diabetic KKAy mice were randomly divided into model (treated with saline), Acarbose (treated with 0.01 mL/g/d acarbose), high-dose (treated with 4.68 g/kg Tangnaikang), medium-dose (treated with 2.34 g/kg Tangnaikang), and low-dose (treated with 1.17 g/kg Tangnaikang) groups (*n* = 13/group). All drugs were administered via gavage once a day for 8 weeks. The control and model groups were administered normal saline (20 mL/kg) once a day for 8 weeks. All mice had free access to water and food during treatment. The fasting plasma glucose (FPG) levels and bodyweights were measured once a week.

### 2.3. Sample Collection

After the final dose of treatment, mice were deprived of food for 10 h, with free access to water. The orbital sinus blood samples were collected for serum isolation. The fecal samples were collected as previously described and stored at –80°C until use [15].

### 2.4. Genomic DNA Isolation

Fecal genomic DNA was isolated using a HiPure Soil DNA kit (Magen, Guangzhou, China) according to the manufacturer's instructions. Briefly, the ground fecal samples were lysed and pretreated with proteinase K and lysozyme, followed by a 2-hour incubation at 55°C. After centrifuging at 12,000 rpm for 5 min, the supernatant was collected, mixed with protein precipitation solution, vortexed for 5 s, and incubated on ice for 5 min. The mixture was centrifuged again at 12,000 rpm for 5 min. The supernatant was collected and mixed with isopropanol, followed by incubation on ice for 20 min. After centrifugation, the precipitation was washed twice with 75% ethanol. The white precipitation was collected after centrifugation, moderately dried, and reconstituted with 50 *µ*L ddH_2_O, followed by incubation at 37°C for 15 min. The diluted genomic DNA was used as the template for 16S rRNA amplification.

### 2.5. 16S rRNA Sequencing

Approximately 30–50 ng genomic DNA was used as the template to amplify the V3-V4 region of the 16S rRNA using the primer sets 5′-CCTACGGRRBGCASCAGKVRVGAAT-3′ (forward) and 5′-GGACTACNVGGGTWTCTAATCC-3′ (reverse). An index adapter sequence was added to the end of the PCR product. The quality and the concentration of the PCR products were determined using an Agilent 2100 bioanalyzer (Agilent Technologies, Palo Alto, CA, USA) and a Qubit 2.0 Fluorometer (Invitrogen, Carlsbad, CA), respectively. The 16s rRNA was sequenced using 250/300 pair-end sequencing on an Illumina MiSeq instrument (Illumina, San Diego, CA, USA). The raw sequence was obtained using MiSeq control software (Illumina).

### 2.6. Gut Microbiota Analysis

The forward and reverse reads obtained from pair-end sequencing were assembled as pairs and filtered by removing the N-containing reads. The clean reads with a length of more than 200 nucleotides were preserved, and the chimeric sequences were removed. The clean reads were then subjected to operating taxonomic unit (OTU) analysis.

The sequence clustering was performed using VSEARCH [16] (1.9.6) at 97% identity, and sequence alignment was conducted using the SILVA 132 database [17]. The species taxonomic analysis on the representative sequences of OTU was conducted using the Ribosomal Database Program classifier Bayesian algorithm [18], followed by counting the community composition of each sample at various taxonomic levels.

Alpha diversity, such as Shannon and Chao1 indexes [19, 20], was calculated using random samples based on OTU analysis, followed by the generation of the dilution curves. The difference in microbial communities between samples was compared using unweighted UniFrac analysis. *β* diversity was analyzed using a principal coordinate analysis (PCoA) plot based on Bray-Curtis distance matrices. The unweighted pair-group method with arithmetic mean phylogenetic tree was constructed using the unweighted pair-group average method of hierarchical clustering.

### 2.7. Statistical Analysis

Data were expressed as the mean ± standard deviation. Statistical analysis was performed using SPSS 22.0 (IBM, Armonk, NY, USA). Differences between groups were compared using one-way analysis of variance, followed by Student's *t*-test, Wilcox rank-sum test, or Tukey's test. A *P* value <0.05 was considered statistically significant.

## 3. Results

### 3.1. The Effects of Tangnaikang on FPG Levels and Bodyweights of KKAy Mice

**Figure 1:**
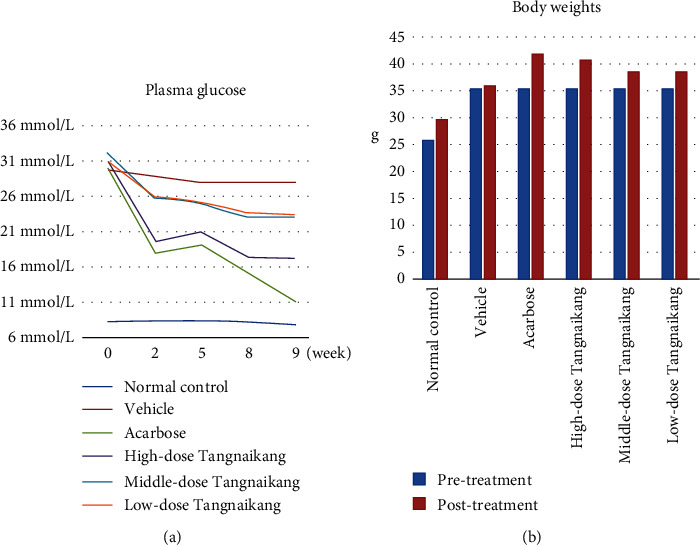
The effects of Tangnaikang treatment on fasting plasma glucose (FPG) levels and bodyweights in KKAy mice. A total of 13 C57BL mice were assigned to the control group and fed a chow diet. A total of 65 male KKAy mice were fed a high-fat diet for 2 weeks, followed by treatment with normal saline (20 mL/kg), Acarbose, high-dose Tangnaikang (4.68 g/kg), medium-dose Tangnaikang (2.34 g/kg), or low-dose Tangnaikang (1.17 g/kg) via gavage once a day for 8 weeks (*n* = 13/group). (a) The curves of FPG levels. (b) Bodyweights before and after 8-week treatment.

To explore the antidiabetic effect of Tangnaikang, we monitored FPG levels and bodyweights of KKAy mice. As shown in Figures 1(a) and 1(b) and Tables 1 and 2, no significant difference was observed in FPG levels before and after treatment in the vehicle group. Except for the vehicle group, other groups exhibited remarkably decreased FPG levels after treatment, with Acarbose showing the maximum effect, followed by high-dose Tangnaikang. Although FPG levels in the medium-dose and low-dose groups exhibited decreasing trends, no statistical significance was observed.

When comparing the bodyweights before and after treatment, we did not observe a significant change in the bodyweights of the vehicle group. Except for those of the vehicle group, the bodyweights of other groups were significantly increased after treatment. No significant difference was observed in the bodyweights of the Acarbose group and the high-dose Tangnaikang group. The bodyweights of the Acarbose group were significantly higher than those of the middle-dose and the low-dose groups. These data suggest that high-dose Tangnaikang exhibits stronger antidiabetic effects than medium- and low-dose Tangnaikang.

### 3.2. The Effect of Tangnaikang Treatment on 16s rRNA in the Gut Microbiota in KKAy Mice

**Figure 2:**
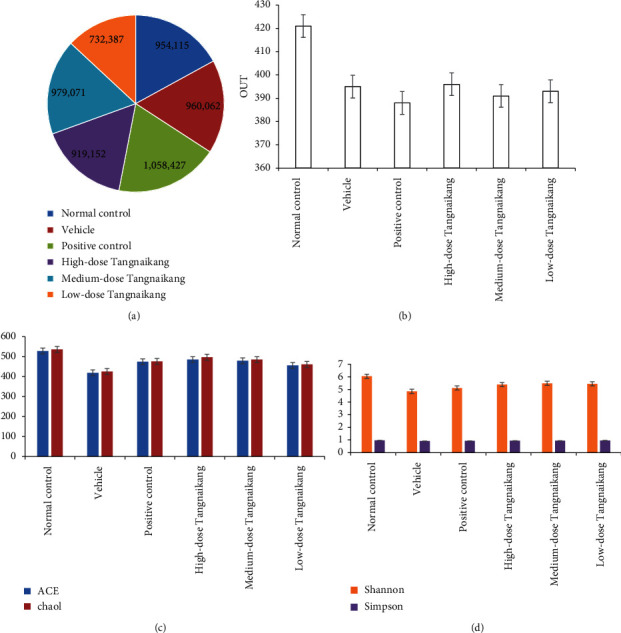
The effects of Tangnaikang treatment on gut microbial abundance and diversity in KKAy mice. Mouse fecal samples were collected at 8 weeks after Tangnaikang treatment. The 16s rRNA sequencing was performed to analyze gut microbial profiles. (a) A pie graph of clean reads in each group. (b) Operational taxonomic units (OTU) analysis. (c) Chao1 and abundance-based coverage estimator were used to evaluate microbial species richness. (d) Shannon and Simpson indexes were used to evaluate microbial diversity.

In the 88 mouse fecal samples, by sequencing the V3-V4 region of the 16S rRNA, we obtained a total of 14756448 reads. After tailoring and modification, we obtained 7378224 high-quality reads, including 954115 (12.93%) reads in the control group, 960062 (13.01%) in the vehicle group, 1058427 (14.35%) in the Acarbose group, 919152 (12.46%) in the high-dose group, 979071 (13.26%) in the medium-dose group, and 732387 (9.92%) in the low-dose group (Figure 2(a)). The number of reads per sample ranged from 47425 to 64808, with an average value of 56416 and a mean length of 454 bp.

### 3.3. OTU Analysis of Gut Microbiota in KKAy Mice

The OTU value indicates the microbial abundance in the mouse gut. The greater the value, the more abundant the microorganisms. OTU is typically assessed by bacterial community abundance index and diversity index. The bacterial community abundance index includes Chao1 and ACE. The larger the index value, the higher the abundance of the bacterial community.

As shown in Figure 2(b) (Supplementary Figure 1) and Table 3, the OTU value of the vehicle group was significantly decreased compared with that of the control group. Compared with saline treatment or Acarbose treatment, Tangnaikang treatment resulted in significant increases in the OTU, ACE, and Chao1 values of KKAy mouse gut microbiota in a dose-dependent manner. This finding suggests that Tangnaikang treatment enhances gut microbial diversity in KKAy mice, with the high-dose group showing the highest microbial diversity and the low-dose group showing a relatively low microbial diversity.

### 3.4. Microbial Community Diversity Indexes

Compared with that of the control group, the Shannon index of the vehicle group was significantly decreased, which was effectively reversed by Tangnaikang or Acarbose treatment (Figure 2(d) and Table 4). No significant differences were observed in the OTU, ACE, and Chao1 values between high-dose and control groups. Thus, T2D significantly impairs gut microbial diversity in KKAy mice.

### 3.5. Comparison of Mouse Gut Microbiota at the Family, Genus, and Class Levels

**Figure 3:**
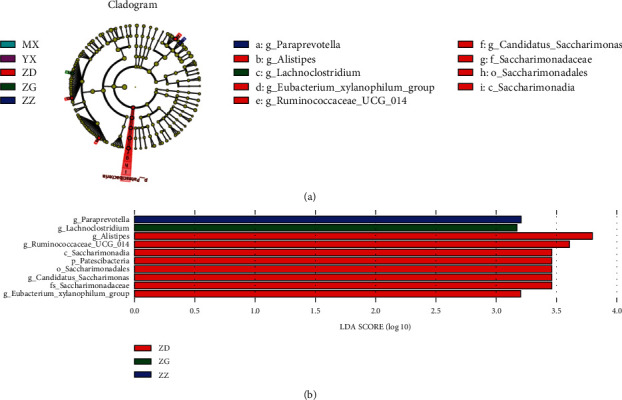
Comparison of the gut microbial taxa among different groups. Taxonomic differences were detected among different groups. (a). The cladogram diagram shows the microbial species with significant differences in the vehicle (blue), Acarbose (purple), low-dose (red), medium-dose (blue), and high-dose (green) groups. The species classification at the levels of phylum, class, order, family, and genus is shown from the inside to the outside. The red, blue, and green nodes in the phylogenetic tree represent differential microbial species in the low-dose, medium-dose, and high-dose groups, respectively. Yellow nodes represent species with no significant difference. (b). Linear discriminative analysis (LDA) effect size (LEfSe) among high-dose (green), medium-dose (blue), and low-dose (red) groups. Species with significant difference have an LDA score >2.0. The length of the histogram represents the LDA score.

In the cladogram diagram (Figure 3(a)), the circles from the inside to the outside indicate the species classification at the levels of phylum, class, order, family, and genus. The diameter of the circle is proportional to the relative abundance. The green, purple, red, blue, and green nodes in the phylogenetic tree represent differential microbial species in the vehicle, Acarbose, low-dose, medium-dose, and high-dose groups, respectively. The histogram of the linear discriminative analysis (LDA) shows the microbial species that were significantly different among groups, with an LDA score >2.0. The length of the histogram represents the LDA score (Figure 3(b)).

As shown in Figures 3(a) and 3(b), in the phylum Patescibacteria, the genera *Candidatus_Saccharimonas*, *Alistipes*, *Eubacterium*, and *Ruminococcaceae* were significantly more abundant in the low-dose group compared with those in other groups. The genera *Paraprevotella* and *Lachnoclostridium* were significantly more abundant in the medium- and high-dose groups, respectively, compared with those in other groups.

### 3.6. The Effect of Tangnaikang Treatment on Gut Microbial Composition in KKAy Mice

**Figure 4:**
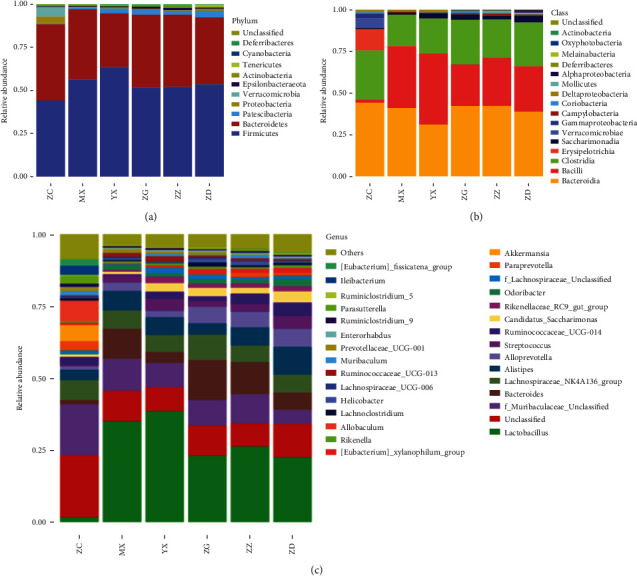
The histogram of relative abundance of bacteria at the phylum (a), class (b), and genus (c) levels.

To further examine the effect of Tangnaikang treatment on gut microbial composition in KKAy mice, we analyzed the relative abundance of gut bacteria at the phylum and genus levels. No significant difference was observed at the phylum level among different groups, and the dominant bacterial phyla in all groups were Firmicute and Bacteroidetes, followed by Proteobacteria, Patescibacteria, Actinobacteria, and Verrucomicrobia (Figure 4(a)).

At the class level (Figure 4(b)), the dominant bacterial classes across the groups were Bacteroidia, Bacilli, Clostridia, and Erysipelotrichia. Compared with the control group, the model group had dramatically increased Erysipelotrichia and Bacilli (*P* < 0.001). The abundance of Erysipelotrichia and Bacilli was significantly decreased in Tangnaikang-treated groups compared with that in the vehicle group (*P* < 0.001), suggesting that Erysipelotrichia and Bacilli are related to T2D.

At the genus level, we selected the top 30 most abundant bacterial genera based on the OTUs for species annotation and generation of a histogram of relative abundance of species. As shown in Figure 4(c), the dominant gut bacterial genera in the control group were Muribaculaceae (17.66%), *Allobaculum* (7.11%), Lachnospiraceae*-NK4A136_group* (6.97%), and *Akkermansia* (5.6%). Compared with the control group, the vehicle group had dramatically increased abundance of *Lactobacillus* (*P* < 0.001), *Bacteroides* (*P* < 0.01), *Alistipes* (*P* < 0.01), and *Streptococcus* (*P* < 0.05). The abundance of Muribaculaceae showed a nonsignificant reduction, whereas the abundance of *Akkermansia* exhibited a significant increase. This suggests that Acarbose restores gut microbial balance by regulating the abundance of *Bacteroides* and *Akkermansia*.

Compared with the vehicle group, Tangnaikang-treated groups had significantly decreased abundance of *Lactobacillus* (*P* < 0.05). Low-dose Tangnaikang significantly reduced the abundance of *f_Muribaculaceae_Unclassified* in KKAy mice compared with saline treatment. On the other hand, Tangnaikang treatment remarkably elevated the abundance of *Akkermansia* and *Allobaculum*. These data suggest that Tangnaikang restores gut microbial balance by regulating the abundance of *Lactobacillus* and *Allobaculum*, which is different from Acarbose.

### 3.7. Principal Component Analysis (PCA) of Mouse Gut Microbiota

**Figure 5:**
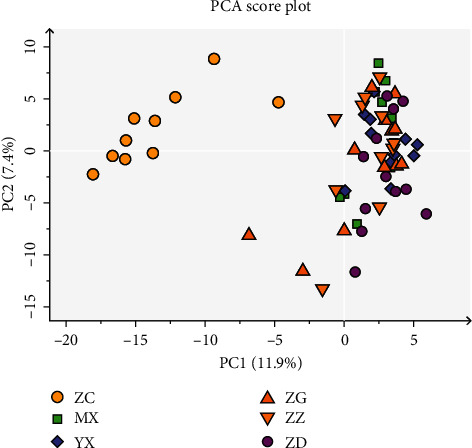
Principal component analysis (PCA) of the gut bacterial community of different groups. The bacterial composition of fecal samples was subjected to PCA. The first two principal components PC1 and PC2 were plotted. The percentage values in parentheses next to PC1 and PC2 represent the percentage of variance explained by each component.

We further performed PCA on bacterial composition in different groups and found that the control group was separated from the other groups (Figure 5), suggesting that the gut microbiota of KKAy mice was significantly different from that of normal control mice. Except for the samples of the control group, the samples of the other groups were partially overlapped or closely clustered. Compared with the samples of the high-dose group, the samples of medium-dose and low-dose groups were closer to those of the model group. This suggests that, compared with the microbial composition of the high-dose group, the microbial composition of the medium-dose or low-dose group is more similar to that of the vehicle group. This indirectly suggests that high-dose Tangnaikang improves gut microbiota of KKAy mice to some extent.

### 3.8. Prediction of Intestinal Flora Function

**Figure 6:**
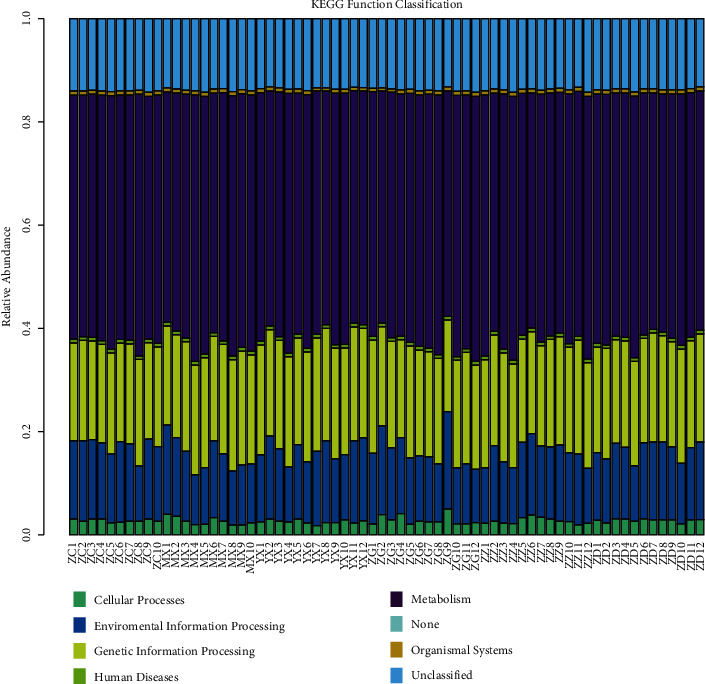
KEGG Function Classification. The abscissa indicates the grouping case, and the ordinate indicates the relative abundance. The legend on the right indicates mean relative abundance of each metabolic pathway across all samples by different colors.

**Figure 7:**
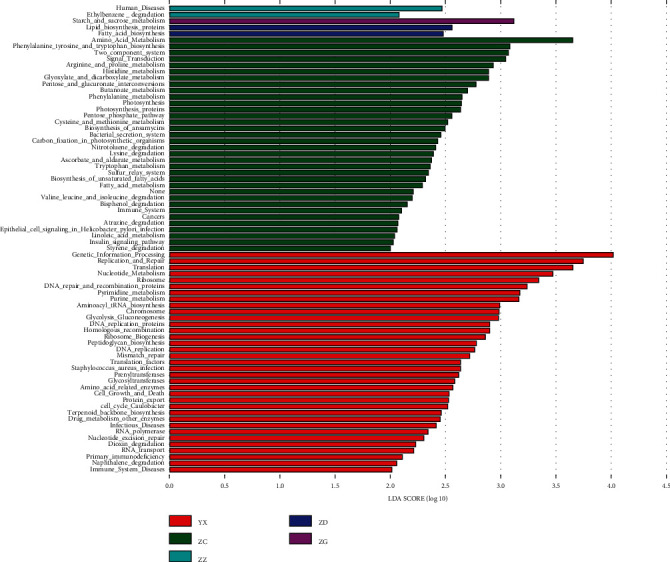
LDA metabolic pathway. Bar graphs of LDA value distribution showing species with different LDA score greater than set values, that is, biomarkers with statistical differences. For species showing significant differences in abundances across groups, the length of the bar graph represents the effect size of significantly different species.

Combined with the KEGG database (Figure 6), the relative abundance of the samples in Metabolism, Environmental Information Processing, Genetic Information Processing, and Human Diseases is significantly increased on this metabolic pathway. In the LDA metabolic pathway (Figure 7), LDA scores for significant acting microbial taxa in different groups were counted, and the biomarkers with statistical differences were shown. The relative abundance of galactose metabolism, carbohydrate, and glucose metabolism function increased significantly in the group with high dose of Tangnaikang, while the group with low dose of Tangnaikang increased significantly in lipid biosynthetic protein, RNA transport function, and fatty acid bioanabolism.

## 4. Discussion

Gut microbiota imbalance contributes to the development of T2D through multiple molecular mechanisms, such as modulating inflammation, affecting gut permeability, and regulating glucose and lipid metabolism, in the mammalian host [21–23]. In this study, we aimed to investigate the involvement of gut microbiota in the antidiabetic effect of Tangnaikang. The results of *in vivo* study showed that, compared with vehicle or low-/medium-dose Tangnaikang treatment, high-dose Tangnaikang treatment remarkably reduced FPG levels while elevating bodyweights in KKAy mice. By analyzing the sequencing results of 16s rRNA from mouse fecal samples, we identified dominant fecal bacteria associated with the antidiabetic role of Tangnaikang. Our results provide new information about the mechanism underlying the therapeutic effect of Tangnaikang on T2D.

In the present study, we evaluated the therapeutic role of Tangnaikang in KKAy mice. We found that high-dose Tangnaikang (4.68 g/kg) outperformed lower doses of Tangnaikang in reducing FPG levels in KKAy mice. Similarly, studies have shown that administration with 3.24 g/kg Tangnaikang significantly reduces blood glucose levels in prediabetic SHR/cp rats and obese Zucker rats [10, 11]. Regarding the changes in bodyweights, Li et al. have shown that Tangnaikang treatment reduces the bodyweights of SHR/cp rats without affecting food consumption starting 3 weeks after treatment [10], whereas Guo et al. have demonstrated that a 4-week Tangnaikang treatment only slightly, but not significantly, reduces the bodyweights of obese Zucker rats [11]. Conversely, our results showed that, compared with vehicle treatment, an 8-week treatment with different doses of Tangnaikang significantly elevated the bodyweights of KKAy mice. The inconsistency among studies may be associated with the differences in the sources of the drug, animal species, and treatment durations. Another possible explanation is that, in this study, HFD-induced weight gain exceeded Tangnaikang-induced weight loss, leading to a net increase in the body weight.

Numerous studies have investigated the association between T2D and the bacterial microbiome; however, reports have shown inconsistency regarding the association of specific taxa with the disease. According to a recent review article that has summarized 42 human studies about the association of the bacterial microbiome with T2D, *Bifidobacterium* and *Bacteroides* are the most reported genera associated with T2D [24]. Consistent with some studies [25–27], our results showed that vehicle-treated model mice had dramatically increased abundance of *Bacteroides* compared with control mice. Interestingly, like Acarbose, low-dose Tangnaikang, but not high- and medium-dose Tangnaikang, significantly reduced the abundance of *Bacteroides* in KKAy mice, suggesting that Tangnaikang regulates the abundance of *Bacteroides* within a certain dose range. Studies have consistently shown a negative association of *Akkermansia* with T2D [24]. We found that *Akkermansia* was one of the dominant bacterial genera in the control mice and almost disappeared in the model mice. However, neither Tangnaikang nor Acarbose showed any effect on the abundance of *Akkermansia*, suggesting that *Akkermansia* may be used as a probiotic supplementation. *Lactobacillus* genus has been considered positively associated with T2D in humans [28–32]. Consistently, our results showed that vehicle-treated model mice had dramatically increased abundance of *Lactobacillus* compared with control mice and that administration of different doses of Tangnaikang significantly reduced the abundance of *Lactobacillus*.

## 5. Conclusions

In this study, we demonstrate that high-dose Tangnaikang reduces FPG levels while elevating bodyweights in KKAy mice. The antidiabetic effect of Tangnaikang is associated with the improvement of the composition and abundance of gut microbiota. Our results provide new insights into the mechanism underlying the therapeutic benefits of Tangnaikang for T2D.

## Figures and Tables

**Table 1:** Comparison of fasting plasma glucose levels in mice ($\bar{x}\pm s$).

Group	*n*	FPG before treatment (mmol/L)	FPG after treatment (mmol/L)
Control	10	8.27 ± 0.85	7.9 ± 0.62
Vehicle	10	29.55 ± 3.95	27.96 ± 5.60^*∗*^
Acarbose	10	29.74 ± 3.73	11.06 ± 3.92^*∗*^^○^
High dose	10	30.65 ± 3.42	17.11 ± 8.01^*∗*^^※^
Medium dose	10	31.74 ± 2.64	22.86 ± 6.02^*∗*^^△^
Low dose	10	30.60 ± 1.62	23.17 ± 6.97^*∗*^^△^

FPG, fasting plasma glucose. Nonparametric test: ^*∗*^*P* < 0.01 versus control group; ^○^*P* < 0.01, Acarbose group versus vehicle group; ^※^*P* < 0.05, vehicle group versus high-dose group; ^△^*P* < 0.01, medium-dose or low-dose group versus Acarbose group; ^▲^*P* < 0.05, medium-dose group versus low-dose group.

**Table 2:** Comparison of the bodyweights of mice ($\bar{x}\pm s$).

Group	n	Bodyweights before treatment (g)	Bodyweights after treatment (g)
Control	10	25.95 ± 1.20	29.71 ± 1.00
Vehicle	10	35.53 ± 1.42	36.28 ± 2.10^*∗*^
Acarbose	10	35.51 ± 1.90	41.84 ± 2.82^*∗*^^○^
High dose	10	35.53 ± 1.57	40.95 ± 1.88^*∗*^^○※^
Medium dose	10	35.41 ± 2.64	38.92 ± 1.58^*∗*^^○△●^
Low dose	10	35.50 ± 2.02	38.66 ± 1.89^*∗*^^○△●^

LSD test: ^*∗*^*P* < 0.01 versus control group; ^○^*P* < 0.01 versus vehicle group; ^△^*P* < 0.01, medium-dose or low-dose group versus Acarbose group; ^※^*P* > 0.05, high-dose group versus Acarbose group; ^●^*P* > 0.05 high-dose group versus medium-dose group.

**Table 3:** Comparison of gut microbial species abundance of mice ($\bar{x}\pm s$).

Group	*n*	OTU	ACE	Chaol
Control	10	421 ± 29	528 ± 33	536 ± 34
Vehicle	10	395 ± 35^b^	419 ± 52^b^	425 ± 55^b^
Acarbose	10	388 ± 65^b^	474 ± 77^ac^	476 ± 79^ac^
High dose	10	396 ± 3^bc^	485 ± 52^ac^	497 ± 52^c^
Medium dose	10	391 ± 40^bc^	479 ± 67^ac^	485 ± 63^ac^
Low dose	10	393 ± 35^bc^	456 ± 39^bc^	461 ± 50^b^

^a^ *P* < 0.05, ^b^*P* < 0.01 versus control group; ^c^*P* < 0.05, ^d^*P* < 0.01 versus vehicle group.

**Table 4:** The effect of Tangnaikang treatment on gut microbial diversity and uniformity in KKAy mice ($\bar{x}\pm s$).

Group	*n*	Shannon	Simpson
Control	10	6.05 ± 0.38	0.96 ± 0.013
Vehicle	10	4.85 ± 0.28^b^	0.91 ± 0.023^b^
Acarbose	10	5.12 ± 0.76^b^	0.92 ± 0.045^b^
High dose	10	5.39 ± 0.36^bd^	0.94 ± 0.015^bc^
Medium dose	10	5.49 ± 0.46^bd^	0.94 ± 0.025^ad^
Low dose	10	5.45 ± 0.38^bd^	0.95 ± 0.015^ad^

^a^ *P* < 0.05, ^b^*P* < 0.01 versus control group; ^c^*P* < 0.05, ^d^*P* < 0.01 versus vehicle group.

## Data Availability

The data that support the findings of this study are available from the corresponding author upon reasonable request.

## Supplementary Materials

Supplementary Materials Supplementary Figure 1: the rarefaction curves of OTUs (the *x*-axis represents the number of valid sequences per sample, the *y*-axis represents OTUs, and each curve represents a different sample).
