# Supplementary material for: Tangnaikang Alleviates Hyperglycemia and Improves Gut Microbiota in Diabetic Mice
Source: Evid Based Complement Alternat Med. 2021 Nov 25;2021:1089176. doi: 10.1155/2021/1089176 (PMC8639253; doi:10.1155/2021/1089176)
Supplement: Supplementary Materials — Supplementary Figure 1: the rarefaction curves of OTUs (the x-axis represents the number of valid sequences per sample, the y-axis represents OTUs, and each curve represents a different sample). [file 1089176.f1.doc]

**
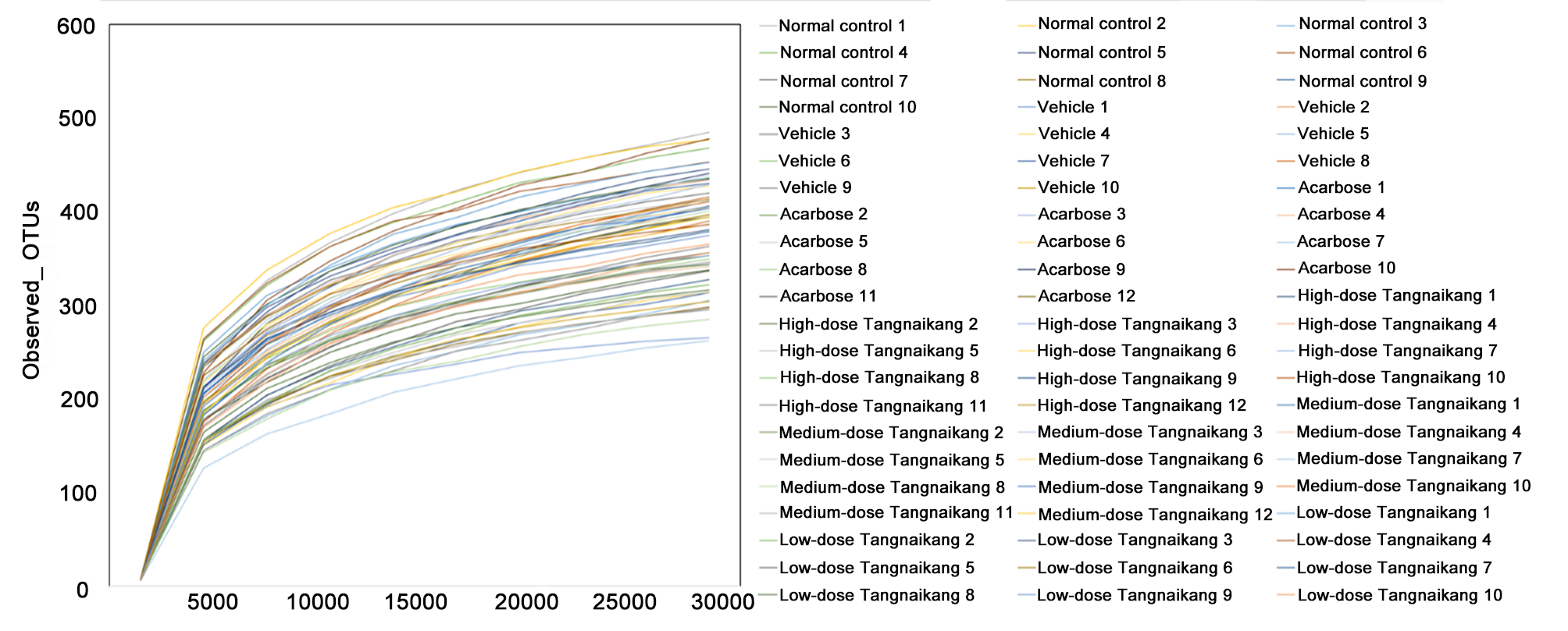
**

**Supplementary Figure 1** The rarefaction curves of OTUs. The x-axis represents the number of valid sequences per sample. The y-axis represents OTUs. Each curve represents a different sample.
